# Six novel *SACS* mutations expand the autosomal recessive spastic ataxia of Charlevoix–Saguenay spectrum

**DOI:** 10.1186/s13023-026-04337-y

**Published:** 2026-04-01

**Authors:** Susumu Ikenoshita, Toshiya Nomura, Haruo Shimazaki, Hiroyuki Uetani, Keiichi Nakahara, Takahiro Okazaki, Michie Imamura, Hironori Mizutani, Aoi Fudo, Yuya Jo, Soichiro Matsubara, Yujiro Higuchi, Toshinori Hirai, Hiroshi Takashima, Mitsuharu Ueda

**Affiliations:** 1 Department of Neurology, Graduate School of Medical Sciences, Kumamoto University, 1-1-1 Honjo, Kumamoto, 860-0811 Japan; 2 Faculty of Health & Medical Care, Saitama Medical University, Saitama, 350-1241 Japan; 3 Department of Neurology, Jichi Medical University, Tochigi, 329-0498 Japan; 4 Department of Diagnostic Radiology, Graduate School of Medical Sciences, Kumamoto University, Kumamoto, 860-0811 Japan; 5 Intractable Neurological Diseases Center, Kumamoto University Hospital, Kumamoto, 860-0811 Japan; 6 Department of Neurology, Kumamoto General Hospital, Yatsushiro, 866-8660 Japan; 7 Department of Neurology and Geriatrics, Kagoshima University Graduate School of Medical and Dental Sciences, Kagoshima, 890-8520 Japan

**Keywords:** Autosomal recessive spastic ataxia of Charlevoix–Saguenay, Charcot–Marie–Tooth disease, *SACS* variants, Hereditary neuropathy

## Abstract

**Background:**

The clinical spectrum of autosomal recessive spastic ataxia of Charlevoix–Saguenay (ARSACS) in Asian populations remains incompletely defined. We aimed to characterize the clinical, radiological, and genetic features of Japanese patients with ARSACS and to expand the mutational and phenotypic spectrum of this disorder.

**Methods:**

We conducted a retrospective case series of patients diagnosed with ARSACS in our department between January 2016 and December 2023. Five patients from four families with biallelic *SACS* variants were identified.

**Results:**

Genetic analysis revealed seven pathogenic *SACS* variants, of which six were novel. Clinical heterogeneity was notable, with age at onset ranging from 1 to 27 years. Four patients showed classical ARSACS-related neuroimaging findings, whereas one patient presented with a Charcot–Marie–Tooth disease (CMT)-mimicking phenotype characterized by predominant peripheral neuropathy, mild cerebellar involvement, and absence of the classical pontocerebellar magnetic resonance imaging features.

**Conclusions:**

The recognition of CMT-mimicking presentations supports considering ARSACS in the differential diagnosis of hereditary peripheral neuropathies, particularly in patients with additional cerebellar, pyramidal, or supportive neuroimaging features.

**Supplementary Information:**

The online version contains supplementary material available at 10.1186/s13023-026-04337-y.

## Background

Autosomal recessive spastic ataxia of Charlevoix–Saguenay (ARSACS) was first described in 1978 as a neurodegenerative disorder characterized by the triad of progressive cerebellar ataxia, lower limb spasticity, and sensorimotor peripheral neuropathy, typically manifesting in early childhood [1]. The identification of the *SACS* gene in 2000 has significantly advanced our understanding of this disorder, leading to an exponential increase in reported cases worldwide and revealing its pan-ethnic distribution [2]. Currently recognized as the second most prevalent early-onset autosomal recessive cerebellar ataxia globally [3], ARSACS remains significantly underdiagnosed because of its phenotypic heterogeneity.

Although ARSACS was first delineated in the Charlevoix–Saguenay region, subsequent studies have identified affected patients worldwide [4]. However, the clinical spectrum and diagnostic clues may be underrecognized in Asian populations, where patients present with variable combinations of spasticity, cerebellar signs, and peripheral neuropathy. Reports from East Asia have described both the “classic” ARSACS phenotype [5] and atypical presentations with predominant neuropathy, which may initially be diagnosed as Charcot–Marie–Tooth disease (CMT) [6]. Systematic characterization of Asian cohorts remains limited; therefore, additional case series are needed to better define the phenotypic boundaries and expand the mutational spectrum of *SACS* in this region.

The diagnostic approach to ARSACS has traditionally relied on characteristic neuroimaging and ophthalmological findings. Typical magnetic resonance imaging (MRI) features include pontine linear hypointensity and superior cerebellar vermis atrophy, while ophthalmological findings include hypermyelinated retinal nerve fibers [7]. However, emerging evidence has expanded the phenotypic spectrum, encompassing cases without spasticity [8–10] and patients with cognitive impairment [11], thereby challenging the traditional diagnostic paradigm.

Since 2016, we have identified five patients with ARSACS from four families who demonstrate remarkable clinical heterogeneity, including adult-onset presentations and a neuropathy-predominant phenotype initially mimicking CMT. In this study, we aimed to characterize the clinical, neuroimaging, and genetic features of ARSACS to expand the phenotypic spectrum of this disorder and provide insights for the diagnostic recognition of non-classical presentations.

## Methods

We conducted a retrospective case series of patients diagnosed with ARSACS in our department between January 2016 and December 2023. Cases were identified through a review of medical records, genetic testing results, and neuroimaging findings obtained during routine clinical practice. At our institution, patients with suspected spinocerebellar degeneration are routinely admitted for comprehensive evaluation; therefore, cases were ascertained through a review of inpatient records. Patients were identified either through targeted *SACS* analysis when ARSACS was clinically suspected based on characteristic neurological and MRI features or through comprehensive genetic testing performed in patients with peripheral neuropathy [12]. We included genetically confirmed patients with biallelic *SACS* variants and sufficient clinical data for detailed analysis. Clinical information, including age at onset, presenting symptoms, disease course, neurological findings, nerve conduction study results, and ophthalmological evaluations, was collected retrospectively from medical records. Brain and cervical spine MRI scans were reviewed for characteristic ARSACS-related findings, including cerebellar atrophy, pontine linear hypointensities, white matter abnormalities, brainstem changes, thalamic signal abnormalities, and spinal cord atrophy. Variants were interpreted according to the American College of Medical Genetics and Genomics guidelines using public databases, including ClinVar and gnomAD.

## Results

### Patient summary

Between January 2016 and December 2023, we systematically evaluated 3,347 neurological inpatients admitted to our department, among whom five patients from four families were diagnosed with ARSACS based on biallelic *SACS* variants. This yielded hospital-based frequencies of 0.15% among all neurological admissions, 1.83% among patients with spinocerebellar degeneration (4/219), and 0.29% among those with peripheral neuropathy (1/348). Six variants were novel, expanding the mutational spectrum of ARSACS in Japan. Clinically, these cases showed a broad phenotypic spectrum ranging from classic ARSACS to neuropathy-predominant presentations that could initially mimic CMT.

### Genetic findings

Biallelic *SACS* variants were identified in all five patients (Tables 1 and 2). The sibling cases from a consanguineous family harbored the same homozygous variant, whereas the remaining patients had compound heterozygous variants. Of the seven variants identified across the cases, six were novel. Detailed variant information and family segregation data are provided in Table 2 and Figure S1 in Additional file 1.

**Table 1:** Clinical characteristics of patients with ARSACS

Preliminary diagnosis group	Case 1	Case 2	Case 3	Case 4	Case 5
	Spinocerebellar degeneration	Spinocerebellar degeneration	Spinocerebellar degeneration	Spinocerebellar degeneration	Hereditary peripheral neuropathy
Sex	M	F	M	F	M
Biallelic *SACS* variants	p.Glu3647Ter/ p.Glu3647Ter	p.Glu3647Ter/ p.Glu3647Ter	p.Arg88Ter/ p.Lys1715Ter	p.Ala720_Gly721insTyr/p.Phe4432Ter *	p.Arg2425Ter/ p.Pro3656Leu
Consanguineous marriage of the parents	+	+	−	−	−
Age of onset (years)	1	7	10	27	Early childhood
Initial symptoms	Gait instability	Gait instability	Gait instability	Gait instability	Impaired running ability
Age at initial presentation (years)	1	24	15	33	44
Age at diagnosis (years)	23	25	16	34	46
Intellectual disability	+	±	+	−	−
Cerebellar ataxia	+	+	+	+	±
Nystagmus	+	+	+	+	−
Dysarthria	+	+	−	−	−
Gait disturbance	+	+	+	+	±
Patellar hyperreflexia	+	+	±	+	+
Babinski sign	+	+	±	+	+
Distal hand atrophy	+	−	+	+	+
Pes cavus	+	+	+	+	+
Nerve conduction studies at diagnosis					
Axonal-demyelinating sensorimotor neuropathy with lower limb predominance	+	+	+	+	+
Median CMAP amplitude (mV)	5.7	2.2	4.4	10.6	5.2
Median MCV (m/s)	49.0	58.9	45.5	38.0	46.1
Median F latency (ms)	40.3	36.4	30.3	30.5	34.1
Median SNAP amplitude (µV)	Absent	Absent	2.3	Absent	18.4
Median SCV (m/s)	Absent	Absent	32.1	Absent	45.5
Peroneal CMAP amplitude (mV)	Absent	Absent	0.4	0.36	Absent
Peroneal MCV (m/s)	Absent	Absent	62.0	23.1	Absent
Sural SNAP amplitude (µV)	Absent	Absent	Absent	Absent	1.2
Sural SCV (m/s)	Absent	Absent	Absent	Absent	27.0
Ophthalmological findings					
Increased visibility of the retinal nerve fiber layer	+	−	+	−	−
Thickening of retinal nerve fibers	+	n/a	+	+	n/a
CNS MRI findings					
Superior cerebellar vermian atrophy	+	+	+	+	−
T2 hypointensities at the ventral pons with lateral hyperintense stripes	+	+	+	+	−
Bilateral symmetrical T2 hyperintensities in the lateral thalamus	−	+	+	+	+
Cervical spinal cord atrophy	+	+	+	+	±

Cases 1 and 2 involved siblings

* Segregation analysis not available

F, Female; M, male; NCS, nerve conduction study; RNF, retinal nerve fibers; pRNFL, peripapillary retinal nerve fiber layer; n/a, not available; ARSACS, autosomal recessive spastic ataxia of Charlevoix–Saguenay; CMAP, compound muscle action potential; MCV, motor conduction velocity; SNAP, sensory nerve action potential; SCV, sensory conduction velocity; CNS, central nervous system; MRI, magnetic resonance imaging

**Table 2:** Seven pathogenic sacsin/*SACS* variants in Japanese ARSACS cases

Sacsin protein variants	SACS gene variants	ClinVar accession no.	ClinVar classification	Allele frequency in gnomAD variants	References on clinical symptoms of variants
p.Arg88Ter	c.262 C > T	VCV000559870.10	Pathogenic	2.48e-6 (4/1,611,046)	n/a
p.Ala720_Gly721insTyr	c.2159_2160insTTA	n/a	n/a	n/a	n/a
p.Lys1715Ter	c.5143 A > T	VCV000917652.10	Pathogenic	8.71e-6 (14/1,607,636)	Ref 3
p.Arg2425Ter	c.7273 C > T	VCV000370415.10	Pathogenic/likely pathogenic	1.18e-5 (19/1,613,212)	n/a
p.Glu3647Ter	c.10939G > T	VCV000458251.8	Pathogenic/likely pathogenic	2.48e-6 (4/1,613,752)	n/a
p.Pro3656Leu	c.10,967 C > T	n/a	n/a	6.20e-7 (1/1,613,888)	n/a
p.Phe4432Ter	c.13294_13295insAA	n/a	n/a	n/a	n/a

Six sacsin/*SACS* variants (p.Arg88Ter, p.Ala720_Gly721insTyr, p.Arg2425Ter, p.Glu3647Ter, p.Pro3656Leu, and p.Phe4432Ter) have not been previously described in the literature in individuals with ARSACS. Two of these variants (p.Ala720_Gly721insTyr and p.Phe4432Ter) are novel. The remaining variants are documented in public databases: three (p.Arg88Ter, p.Arg2425Ter, and p.Glu3647Ter) are present in ClinVar, and four (p.Arg88Ter, p.Arg2425Ter, p.Glu3647Ter, and p.Pro3656Leu) are found in gnomAD

We used NG_012342.1 and NM_014363.6 as reference sequences

n/a, not available; ARSACS, autosomal recessive spastic ataxia of Charlevoix–Saguenay

### Clinical Characteristics

Clinical presentations were heterogeneous. Age at onset ranged from childhood to adulthood, and gait disturbance was the predominant initial manifestation. Cerebellar ataxia and peripheral neuropathy were present in all patients, whereas cognitive involvement and pyramidal signs varied across cases. One patient exhibited a neuropathy-predominant phenotype with relatively mild cerebellar findings, initially resembling CMT.

### Electrophysiological and ophthalmological findings

Nerve conduction studies consistently demonstrated sensorimotor neuropathy with mixed axonal and demyelinating features, predominantly affecting the lower limbs. Ophthalmological evaluation revealed ARSACS-associated retinal abnormalities in several patients, including increased visibility or thickening of the retinal nerve fiber layer (RNFL); however, no patient reported significant visual symptoms.

### Neuroimaging findings

Neuroimaging showed substantial heterogeneity. Four patients exhibited typical ARSACS-related MRI features, including superior vermian atrophy, characteristic pontine signal abnormalities, and cervical spinal cord atrophy. In contrast, one patient lacked the typical pontocerebellar findings despite a confirmed molecular diagnosis and presented with a neuropathy-predominant phenotype that initially resembled CMT. Bilateral lateral thalamic T2 hyperintensities were observed in several patients, including the atypical case (Figure S2 in Additional file 1).

## Discussion

In this retrospective case series, we identified five patients with ARSACS harboring seven *SACS* variants, six of which were novel. Notably, one patient presented with a neuropathy-predominant phenotype that initially resembled CMT and lacked classical MRI features, thereby expanding the recognized clinical spectrum and suggesting potential underdiagnosis of ARSACS among Japanese patients with hereditary neuropathies.

Our cases demonstrated notable phenotypic heterogeneity. Age at onset ranged from 1 to 27 years, with Case 5 representing a particularly atypical presentation, characterized by predominant peripheral neuropathy with minimal cerebellar involvement and absence of the pathognomonic MRI features [7, 13]. This variability extends previous Japanese reports describing atypical presentations, including patients lacking spasticity [8] or prominent retinal myelinated fibers [14]. These findings suggest that ARSACS in Japanese patients may deviate substantially from classical diagnostic criteria.

The frequency of ARSACS among peripheral neuropathy cases in our cohort (0.29%) closely aligns with the 0.3% prevalence reported by Pi et al. among Korean families with suspected CMT [15]. However, an important distinction should be noted. Although the Korean patients were identified in a CMT cohort, they showed concurrent cerebellar ataxia and spasticity and retained characteristic brain MRI features, including superior vermian atrophy and pontine signal abnormalities [15]. In contrast, Case 5 in the present study lacked these classical imaging findings entirely. This difference suggests that reliance on traditional clinical and imaging criteria may contribute to underrecognition of ARSACS in Asian populations. Indeed, atypical ARSACS cases lacking overt spasticity, as well as cases initially diagnosed as CMT, have been reported from Japan and China, suggesting that such phenotypes may be relatively frequent in East Asian populations (Table S1 in Additional file 1). Neuroimaging is a critical diagnostic tool in ARSACS [7, 13]. While four patients exhibited the pathognomonic combination of superior vermian atrophy and pontine linear hypointensities, Case 5 lacked these findings despite a confirmed genetic diagnosis. Notably, the “thalamic rim sign,” characterized by bilateral thalamic hyperintensities and reported in 100% of cases in previous series [16], was preserved even in Case 5, suggesting its value as an additional diagnostic marker [17] when classical features are absent. These findings highlight the need to reconsider diagnostic algorithms that rely heavily on pontine and vermian abnormalities.

ARSACS should not be regarded as a purely hereditary neuropathy but rather as a multisystem disorder characterized by variable combinations of cerebellar ataxia, pyramidal signs, and peripheral neuropathy. This overall neurological pattern, which has been well-described in previous series, may help distinguish ARSACS from CMT even when neuropathic features predominate. Nerve conduction studies in ARSACS may demonstrate demyelinating features, including slowed conduction velocities and prolonged distal latencies; however, these abnormalities are not necessarily as severe or as uniform as those typically observed in classical CMT1, and some patients may even show mixed or predominantly axonal features. Therefore, demyelinating findings on nerve conduction studies should be interpreted within the broader clinical context, particularly when cerebellar, pyramidal, retinal, or characteristic neuroimaging features are present. Recent optical coherence tomography (OCT) studies have demonstrated that RNFL changes in ARSACS represent true axonal hypertrophy rather than hypermyelination [18–20], suggesting primary neuronal involvement. The dissociation between structural retinal abnormalities and preserved visual function observed in our patients supports RNFL thickening as a potential diagnostic biomarker, emphasizing the importance of OCT assessment even in asymptomatic patients.

The substantial diagnostic delay observed in our cases underscores persistent challenges in ARSACS recognition, which now encompasses presentations from classic early-onset forms to adult-onset cases with minimal cerebellar involvement [21]. Clinically, ARSACS should be considered in the differential diagnosis of both hereditary ataxias and peripheral neuropathies, regardless of age or classical neuroimaging features. The combination of demyelinating neuropathy without nerve enlargement [22], thalamic hyperintensities, and RNFL thickening may facilitate diagnosis even when pontine and vermian abnormalities are absent.

Some limitations should be acknowledged. The cross-sectional design precludes assessment of disease progression, and the small sample size limits definitive genotype–phenotype correlations. Whether the classical and CMT-mimicking phenotypes reflect distinct pathogenic mechanisms remains unclear. Given that sacsin is a large multidomain protein involved in mitochondrial dynamics, cytoskeletal organization, and protein quality control, both phenotypes likely represent variable manifestations of sacsin dysfunction influenced by variant type, residual protein function, and genetic background. Future prospective studies incorporating functional analyses of novel variants and validation of emerging biomarkers in larger multicenter cohorts are needed.

## Conclusions

Our identification of six novel *SACS* variants highlights the phenotypic heterogeneity in Japanese patients with ARSACS, including CMT-mimicking presentations without characteristic MRI features. These findings expand both the genetic and clinical boundaries of ARSACS, supporting its consideration in the differential diagnosis of hereditary ataxias and peripheral neuropathies, particularly when suggestive clinical or neuroimaging features are present.

## Supplementary Information

Below is the link to the electronic supplementary material.

Supplementary Material 1

## Data Availability

Due to privacy and ethical restrictions, the datasets supporting the current study cannot be publicly shared. Data are available from the corresponding author upon reasonable request and with appropriate institutional approvals.

## Abbreviations

ARSACS: Autosomal recessive spastic ataxia of Charlevoix–Saguenay
CMAP: Compound muscle action potential
CMT: Charcot–Marie–Tooth disease
MRI: Magnetic resonance imaging
OCT: Optical coherence tomography
RNFL: Retinal nerve fiber layer
SNAP: Sensory nerve action potential
