# Supplementary material for: Six novel SACS mutations expand the autosomal recessive spastic ataxia of Charlevoix–Saguenay spectrum
Source: Orphanet J Rare Dis. 2026 Apr 1;21:196. doi: 10.1186/s13023-026-04337-y (PMC13169537; doi:10.1186/s13023-026-04337-y)
Supplement: Supplementary file 1 — Supplementary Material 1 [file 13023_2026_4337_MOESM1_ESM.docx]

**Additional file 1**

**
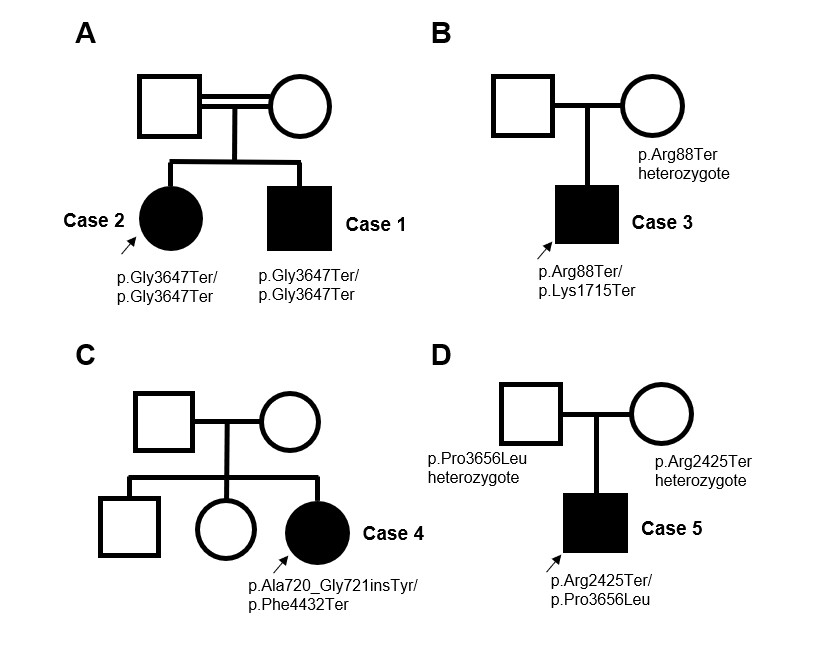
**

**Figure S1. Pedigrees and ARSACS gene mutations in five Japanese cases from four families.**

The pedigrees (A–D) show family relationships and genetic findings in patients with ARSACS. Black symbols indicate individuals with ARSACS, and arrows denote probands. Family A includes siblings (Cases 1 and 2) harboring homozygous mutations (c.10939 G>T). Family B has a proband (Case 3) with compound heterozygous mutations (c.262 C>T and c.5143 A>T). Family C includes Case 4 with compound heterozygous mutations (c.2159_2160 insTTA and c.13294_13295 insAA). Family D includes Case 5 with compound heterozygous mutations (c.7273 C>T and c.10967 C>T). Square symbols represent males, circular symbols represent females, and open symbols indicate unaffected individuals.

ARSACS, autosomal recessive spastic ataxia of Charlevoix–Saguenay.


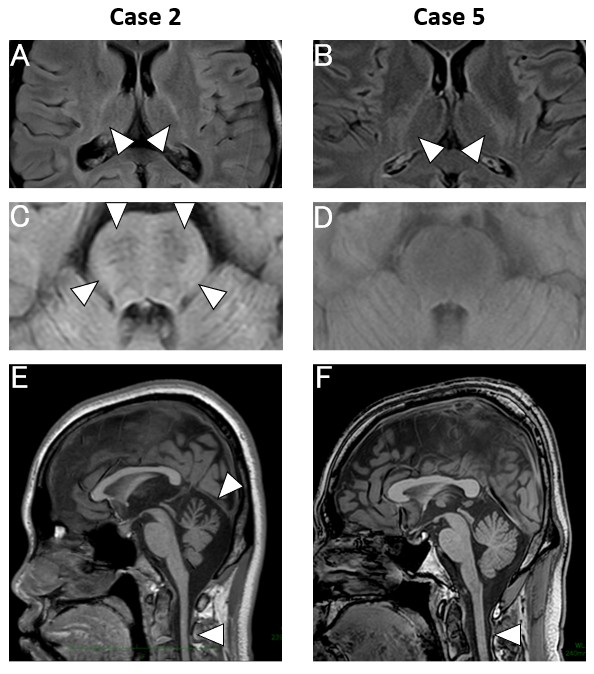


**Figure S2. Illustrative comparison of classical and atypical brain MRI findings in two patients with ARSACS.**

Case 2 (left panels) shows classical ARSACS-related MRI abnormalities, whereas Case 5 (right panels) shows a more atypical imaging profile. A–B: Axial FLAIR images demonstrate bilateral symmetrical hyperintensities in the lateral thalami (arrowheads) in both cases. C–D: Axial T2-weighted images at the pontine level show ventral pontine hypointensities with lateral hyperintense stripes in Case 2 (arrowheads in C), whereas these abnormalities are not evident in Case 5 (D). E–F: Sagittal T1-weighted images show superior cerebellar vermian atrophy (arrow in E) and cervical spinal cord atrophy (arrowheads in E) in Case 2, whereas Case 5 shows preserved cerebellar vermis and only mild cervical spinal cord atrophy (arrowheads in F).

ARSACS, autosomal recessive spastic ataxia of Charlevoix–Saguenay; MRI, magnetic resonance imaging; FLAIR, fluid-attenuated inversion recovery.

**Table S1. Atypical ARSACS cases in East Asia**

| Country  Age/Sex | Neuropathy | Cerebellar ataxia | Spasticity | Cerebellar atrophy | Pontine linear hypointensity | CMT-like | Reference |
| --- | --- | --- | --- | --- | --- | --- | --- |
| Japan  30/M | Present | Present | Absent | Yes | n/a | No | [1] |
| Japan  54/M | Present | Present | Absent | Yes | Yes | No | [2] |
| Japan  44/F | Present | Present | Absent | Yes | Yes | No | [3] |
| China  12/F | Present | minimal | Present | Yes | Yes | Yes | [4] |
| China  58/M | Present | Present | Absent | Yes | Yes | Yes | [5] |

ARSACS, autosomal recessive spastic ataxia of Charlevoix–Saguenay; CMT, Charcot–Marie–Tooth disease; F; Female, M; Male, n/a; not available.

[1] Shimazaki H, Takiyama Y, Sakoe K, Ando Y, Nakano I. A phenotype without spasticity in sacsin-related ataxia. Neurology. 2005;64:2129-31.

[2] Miyatake S, Miyake N, Doi H, Saitsu H, Ogata K, Kawai M, et al. A novel SACS mutation in an atypical case with autosomal recessive spastic ataxia of Charlevoix-Saguenay (ARSACS). Intern Med. 2012;51:2221-6.

[3] Aida I, Ozawa T, Fujinaka H, Goto K, Ohta K, Nakajima T. Autosomal recessive spastic ataxia of Charlevoix-Saguenay without spasticity. Intern Med. 2021;60:3963-7.

[4] Liu L, Li XB, Zi XH, Shen L, Hu ZhM, Huang ShX, et al. A novel hemizygous SACS mutation identified by whole exome sequencing and SNP array analysis in a Chinese ARSACS patient. J Neurol Sci. 2016;362:111-4.

[5] Chen M, Wang X, Ye X, Fang H, Wu Z, Yang J, et al. Novel SACS variants not recorded in ClinVar identified in a Chinese patient with late-onset hereditary neuropathy: a case report and literature review. Cerebellum. 2025;24:160.
